# Human papillomavirus awareness and prevention practices among patients with Mayer–Rokitansky–Küster–Hauser syndrome after neovagina reconstruction: a cross-sectional study

**DOI:** 10.1186/s12905-026-04704-8

**Published:** 2026-07-25

**Authors:** Yi Zhai, Jia Kang, Lan Zhu, Na Chen

**Affiliations:** 1 National Clinical Research Center for Women’s Health and Obstetric and Gynecologic Diseases, Department of Obstetrics and Gynecology, Peking Union Medical College Hospital, Chinese Academy of Medical Sciences & Peking Union Medical College, Beijing, China; 2 State Key Laboratory for Complex Severe and Rare Diseases, Chinese Academy of Medical Sciences & Peking Union Medical College, No. 1 Shuaifuyuan, Beijing, 100005 China

**Keywords:** MRKH syndrome, HPV, Neovaginal reconstruction, HPV vaccine, Preventive awareness

## Abstract

**Background:**

Patients with Mayer–Rokitansky–Küster–Hauser syndrome (MRKH) who undergo neovagina reconstruction remain at risk of Human papillomavirus infection (HPV), yet evidence regarding their awareness of HPV-related health risks and engagement in preventive behaviours is limited. This study aimed to assess HPV-related awareness, preventive practices and behavioural factors among Chinese patients with MRKH syndrome after neovagina reconstruction, and to describe HPV screening and vaccination uptake in this population.

**Methods:**

A cross-sectional questionnaire-based study was conducted at a tertiary referral hospital in Beijing, China. A total of 157 patients with MRKH syndrome who had undergone neovagina reconstruction completed validated questionnaires assessing HPV awareness, preventive practices and behavioural factors. In addition, outpatient clinical records of 1,756 patients with MRKH syndrome attending specialist care between 2012 and 2024 were retrospectively reviewed to obtain information on HPV testing and ThinPrep cytologic test (TCT) results where available. Descriptive statistical analyses were performed to summarise awareness levels, behavioural characteristics and preventive practice uptake.

**Results:**

Most participants demonstrated relatively high awareness of HPV-related health risks: 82.8% recognised their susceptibility to HPV infection and 75.8% expressed support for HPV vaccination. However, preventive practices remained limited. Only 11.5% of participants reported having undergone HPV screening, and 18.5% had received HPV vaccination, indicating a substantial gap between awareness and preventive action. Engagement with routine gynaecological follow-up was generally low. Among those who underwent HPV testing, HPV positivity was detected in a small proportion of patients, with observed rates ranging from 7.4% to 11.1%.

**Conclusions:**

Despite relatively high awareness of HPV and its prevention, Chinese patients with MRKH syndrome after neovagina reconstruction showed markedly low uptake of HPV vaccination and limited engagement with HPV testing. These findings highlight an unmet need for accessible HPV vaccination, neovaginal HPV risk education, and tailored sexual health education for this rare and underserved population. Further research is warranted to identify barriers to preventive care and to develop effective interventions to improve HPV prevention practices among women with MRKH syndrome.

**Supplementary Information:**

The online version contains supplementary material available at https://doi.org/10.1186/s12905-026-04704-8.

## Background

Mayer–Rokitansky–Küster–Hauser (MRKH) syndrome is characterised by the congenital absence of the uterus and vagina despite normal ovarian function, affecting approximately 1 in 4,500–5,000 female live births [1]. The primary therapeutic approach to restoring sexual function in cases of neovagina reconstruction is surgical or nonsurgical, with techniques ranging from dilation methods to surgical interventions such as peritoneal vaginoplasty, biologic graft implantation or other vaginoplasty surgery [2, 3]. Whilst the efficacy of these procedures in achieving satisfactory anatomical and functional outcomes has been demonstrated, emerging evidence suggests that neovaginal tissues remain susceptible to human papillomavirus (HPV) infections, raising concerns about long-term oncogenic risks [4–6].

HPV is a sexually transmitted pathogen associated with benign and malignant lesions of the lower genital tract [7]. For Chinese MRKH patients, the absence of a cervix obstructed clinical attention to the neovagina and vulva as potential sites for HPV-related pathologies. Because in China, HPV vaccines are commonly referred to as “cervical cancer vaccines”, which may contribute to the misconception that HPV prevention is less relevant for women without a cervix. The high-risk HPV types are linked not only to cervical cancer but also to several other malignancies, including anal and oropharyngeal cancers, thereby representing a substantial public health concern [8]. It is worthy of note that Frega et al. conducted a subsequent study with 33 MRKH patients who were positive for HPV over a period of five years, and reported that 28% of these patients experienced recurrent lesions, including precancerous vaginal intraepithelial neoplasia (VAIN) and adenocarcinoma [6]. In contrast, Rall et al. [5] observed a vaginal HPV prevalence of 22% in 186 patients with MRKH syndrome following laparoscopic neovaginoplasty. This prevalence was commensurate with that of cervical infection rates in the general population, yet exhibited minimal cytological abnormalities. These findings underscore substantial knowledge gaps concerning HPV epidemiology in this population, particularly the impact of surgical techniques, sexual behaviours, and preventive measures.

A unique challenge for MRKH patients is their reproductive anatomy: without a uterus, pregnancy is impossible, which could lead to reduced use of contraceptives such as condoms. This behavioural pattern may increase HPV exposure, yet systematic data on HPV transmission dynamics and persistence in neovaginal tissues is scarce. Furthermore, the impact of neovaginal epithelial characteristics, which are influenced by surgical methods such as skin grafts versus peritoneum, on HPV susceptibility and lesion progression is not well understood. Despite increasing advocacy for HPV vaccination and routine surveillance [5], evidence-based guidelines for HPV prevention and management in MRKH patients are lacking.

Given the absence of data from China, this study aimed to examine HPV-related awareness, preventive practices and behavioural factors among Chinese patients with MRKH syndrome after neovagina reconstruction, while descriptively reporting available HPV screening outcomes to inform long-term gynaecological care.

## Methods

### Study Phase 1: cross-sectional questionnaire survey

#### Questionnaires setting and patients

From December 2024 to January 2025, we conducted a voluntary questionnaire survey of women with MRKH syndrome treated at the Department of Obstetrics and Gynaecology, Peking Union Medical College Hospital (PUMCH). Participants were recruited from an MRKH support group linked to our centre and managed through a dedicated WeChat group. Established by Dr. Lan Zhu and Dr. Na Chen, this is the largest MRKH-focused support network in China, with 980 verified members by January 2025 and new patients joining weekly after diagnosis in Dr. Zhu’s clinic. To ensure privacy and data quality, membership is restricted to individuals with a confirmed MRKH diagnosis.

Inclusion criteria were a verified MRKH diagnosis—defined as primary amenorrhoea, a 46,XX karyotype, normal female hormone levels, a primordial uterus on MRI, and absence of a vagina on gynaecological examination—and prior neovaginal reconstruction using dilation, peritoneal vaginoplasty, biological grafts, or other techniques. Patients with vaginal atresia from other causes, including complete androgen insensitivity syndrome (CAIS) or congenital cervical atresia, were excluded and not admitted to the WeChat group.

#### Questionnaires data collection

Structured questionnaires were used to collect demographic information, surgical history, sexual behaviours (number of partners, condom use and frequency), and HPV vaccination and screening status (Supplementary Material 1). An introductory letter explaining the study’s purpose, voluntary participation and anonymity protections was electronically distributed to all group members. Participants completed the questionnaire anonymously through a secure online platform, ensuring ethical compliance, minimising recruitment bias and protecting confidentiality.

**Fig. 1:**
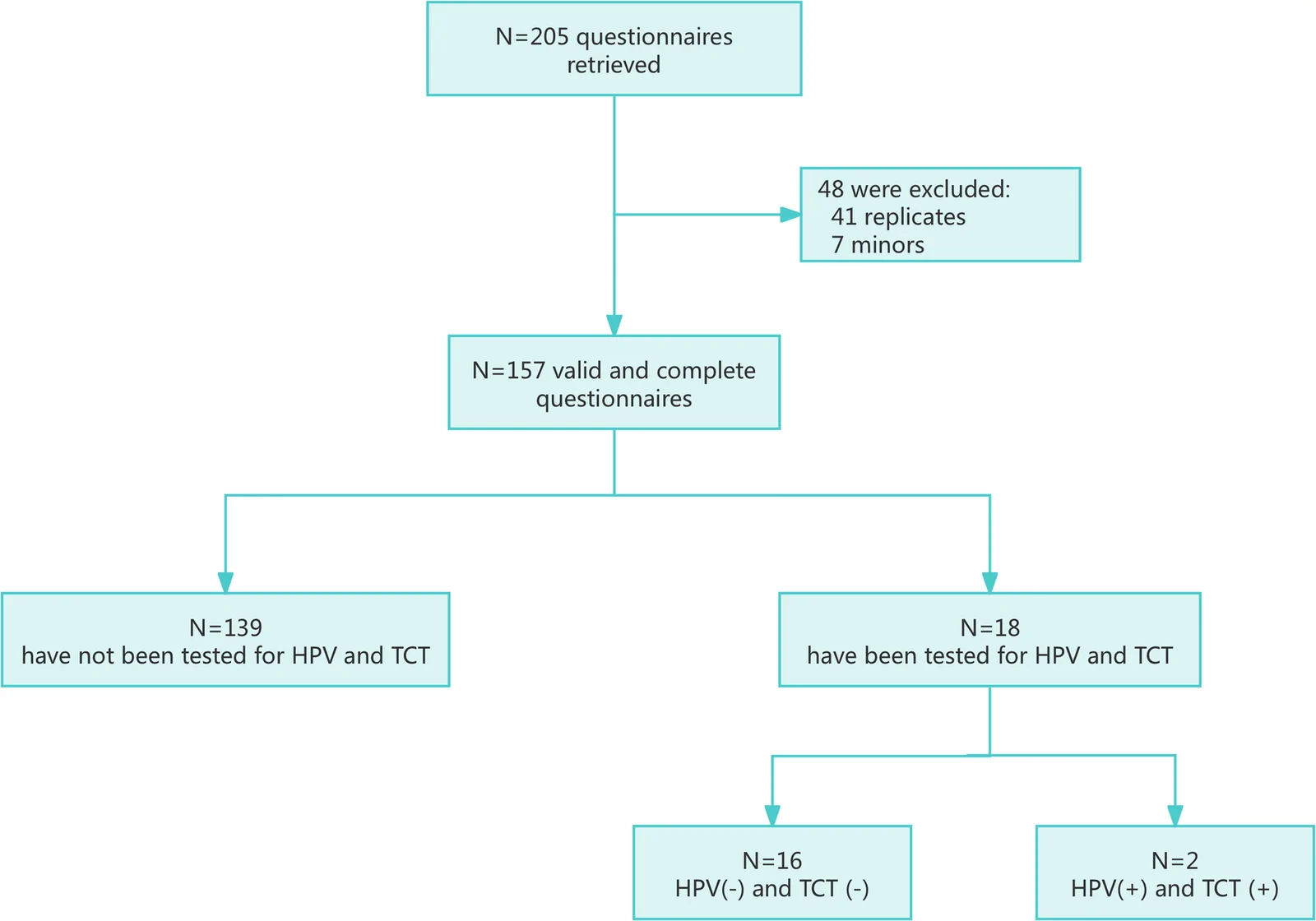
Flow chart of the questionnaire study

A total of 205 questionnaires were received. After excluding 41 duplicates and seven responses from underage participants, 157 fully completed questionnaires were included in the final analysis (Fig. 1).

### Study Phase 2: retrospective outpatient electronic health record review

#### Outpatient data collection

**Fig. 2:**
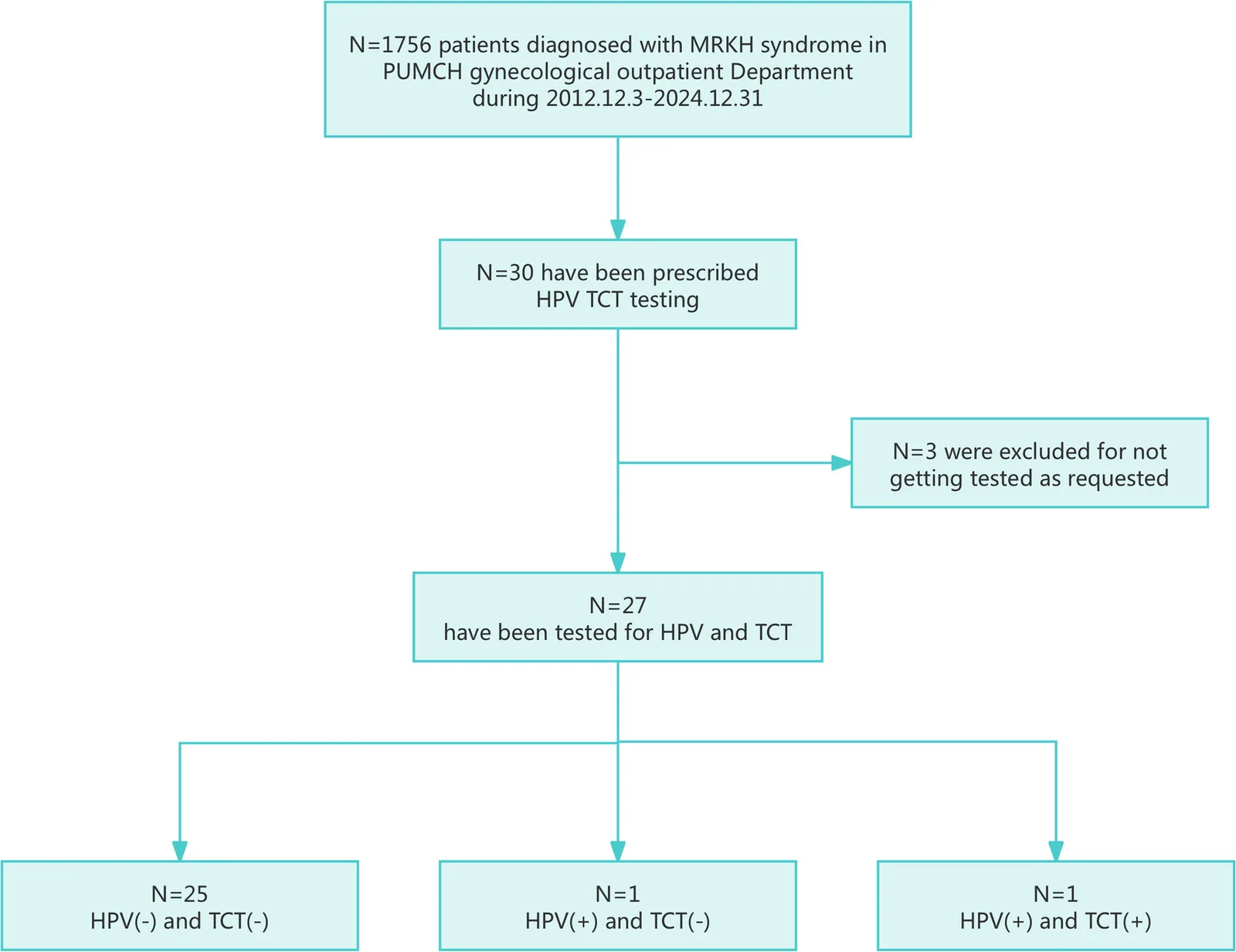
Flow chart of the hospital outpatient data

The second cohort included outpatients diagnosed with MRKH syndrome from 3 December, 2012 to 31 December, 2024 at the Gynecological Outpatient Department of PUMCH. Of the 30 patients prescribed for testing, 27 completed it, while 3 were excluded for non-compliance. The 27 MRKH patients who completed the HPV and TCT tests were considered valid (Fig. 2).

#### TCT and HPV test

Exfoliated cells from the apex of the neovagina were collected using a ThinPrep sampling brush, stored in Preserve Cyt solution and processed with the ThinPrep® 2000 system. Cytology was evaluated according to the 2014 Bethesda System.

High-risk HPV (hr-HPV) testing was performed using the same liquid-based cytology specimen. Hr-HPV DNA was analysed with the Cobas 4800 system (Roche, USA), which uses PCR to detect HPV 16, HPV 18 and 12 additional high-risk types (31, 33, 35, 39, 45, 51, 52, 56, 58, 59, 66 and 68).

### Statistical analysis

The baseline characteristics of patients were described using the median and interquartile range (IQR) for age, while other variables were described using total counts and percentages. Data analysis was performed using SPSS v26.0.

## Results

### Study Phase 1: cross-sectional questionnaire survey

#### Baseline characteristics of questionnaire survey patients

A total of 157 MRKH patients completed the questionnaire. The median age was 27 years (IQR 23–31). Vaginal dilation was the most common treatment (92 patients, 58.6%), followed by peritoneal vaginoplasty (33, 21.0%) and biological grafts (28, 17.8%). Other techniques—such as intestinal, amniotic membrane, oral mucosa or maternal vaginal mucosa grafts—were used in only a few cases (< 1% each).

Regarding lifestyle and sexual behaviour, 9 patients (5.7%) reported smoking. Sexual activity was reported by 106 patients (67.5%), of whom 36 (34.0%) consistently used condoms and 70 (66.0%) used them inconsistently or not at all. Among sexually active patients, 33 (31.1%) had been active for < 1 year and 28 (26.4%) for > 5 years. Intercourse frequency ranged from < 1/month (19 patients, 17.9%) to 3–5 times/week (3 patients, 2.8%). Additionally, 34 patients (32.1%) reported ≥ 2 sexual partners, including 14 (13.1%) with ≥ 3 partners (Table 1).

**Table 1:** Baseline characteristic of patients included in the questionnaire survey

	N (%) or Median (IQR)
**Age, y (*n* = 157)**	27 (23–31)
**Methods of vaginal reconstruction (*n* = 157)**	
Vaginal dilation	92 (58.6%)
Peritoneal vaginal reconstruction	33 (21.0%)
Biological graft vaginal reconstruction	28 (17.8%)
Intestinal neovagina construction	1 (0.6%)
Amniotic membrane vaginal reconstruction	1 (0.6%)
Oral mucosa vaginal reconstruction	1 (0.6%)
Maternal vaginal mucosal transplantation	1 (0.6%)
**History of Smoking (*n* = 157)**	
Yes	9 (5.7%)
No	148 (94.3%)
**History of sexual activity (*n* = 157)**	
Yes	106 (67.5%)
No	51 (32.5%)
**Sexual activity with condoms (*n* = 106)**	
Yes	36 (34.0%)
No	70 (66.0%)
**Sexual activity duration (*n* = 106)**	
< 1 year	33 (31.1%)
1 ~ 3 year	23 (21.7%)
3 ~ 5 year	22 (20.8%)
> 5 year	28 (26.4%)
**Frequency of sexual intercourse (*n* = 106)**	
< 1 time/month	19 (17.9%)
1 ~ 3 times/month	41 (38.7%)
1 ~ 2 times/week	41 (38.7%)
3 ~ 5 times/week	3 (2.8%)
> 5 times/week	2 (1.9%)
**Number of sexual partners (*n* = 106)**	
1	73 (68.8%)
2	19 (17.9%)
3	10 (9.4%)
4	1 (0.9%)
5	3 (2.8%)
**HPV screening (*n* = 157)**	
No	139 (88.5%)
Yes	18 (11.5%)
**HPV-positive (*n* = 18)**	2 (11.1%)
**Cytological abnormalities (*n* = 18)**	2 (11.1%)

#### HPV screening and positive rates in the questionnaire survey

Only 18 patients (11.5%) had undergone HPV screening. Among them, two (11.1%) tested positive for high-risk HPV (Table 1). Both positive cases showed cytological abnormalities—one HSIL and one LSIL—labelled as QO-Patients 1 and Q-Patients 2 (Table 3). These findings suggest that limited screening may lead to underdiagnosis of HPV-related pathology in this population.

#### HPV vaccination and awareness in the questionnaire survey

Although 130 participants (82.8%) recognised that MRKH patients are susceptible to HPV infection and 119 (75.8%) acknowledged the necessity of vaccination, uptake of the vaccine remained low. Only 29 participants (18.5%) had received an HPV vaccine, with the majority opting for the 9-valent vaccine (16/29; 55.2%). Among those who were not vaccinated (128/157, 81.5%), 44 (34.4%) said they did not want to receive the vaccine, while 52 (40.6%) said they would prefer the 9-valent vaccine (Table 2).

**Table 2:** Awareness for HPV infection and vaccination among patients with MRKH syndrome included in the questionnaire survey

	N (%)
**Do you think MRKH patients are susceptible to HPV infection? (*n* = 157)**	
Yes	130 (82.8%)
No	27 (17.2%)
**Do you think it is necessary for MRKH patients to receive the HPV vaccine? (*n* = 157)**	
Yes	119 (75.8%)
No	38 (24.2%)
**Have you received the HPV vaccine? (*n* = 157)**	
Yes, bivalent HPV vaccine	6(3.8%)
Yes, quadrivalent HPV vaccine	7 (4.5%)
Yes, 9-valent HPV vaccine	16 (10.2%)
No	128 (81.5%)
**Are you willing to receive the HPV vaccine in the future? (*n* = 128)**	
Yes, I am willing to receive the bivalent HPV vaccine	14 (10.9%)
Yes, I am willing to receive the quadrivalent HPV vaccine	18 (14.1%)
Yes, I am willing to receive the 9-valent HPV vaccine	52 (40.6%)
No	44 (34.4%)

### Study phase 2: retrospective outpatient electronic health record review

#### HPV infection rate based on outpatient data

To determine the rate of HPV infection in MRKH syndrome patients in a larger cohort, we investigated the HPV and TCT results of patients who attended the PUMCH Outpatient Department between 3 December 2012 and 31 December 2024. Alarmingly, of the 1,756 patients, only 30 were prescribed HPV and TCT tests, and three of these patients did not undergo testing as required. The remaining 27 patients were adults with a median age of 31 (interquartile range: 26–35). The HPV positivity rate was 7.4% (2/27), with one patient developing HSIL (QO-Patient 1 also mentioned in the questionnaire, Table 3), and the other (O-Patient 3, Table 3) not showing cytological abnormalities after HPV infection and not appearing in the questionnaire survey.

**Table 3:** Clinical characteristics of all HPV-positive patients

	**QO-Patient 1**^**a**^	**Q-Patient 2**	**O-Patient 3**
Age (y)	34	33	32
Positive HPV type	High-risk HPV types other than 16 and 18	68, 81	High-risk HPV types other than 16 and 18
Cytology	HSIL^b^	LSIL^c^	Within normal limits
Methods of vaginal reconstruction	Maternal vaginal mucosal transplantation	Vaginal dilation	Biological graft vaginal reconstruction
History of sexual activity	Yes	Yes	Yes
Sexual activity duration	9 years	5 years	5 years
Sexual activity with condoms	No	Yes	No
Frequency of sexual intercourse	3 ~ 5 times/week	< 1 time/month	1 ~ 3 times/month
Number of sexual partners	2	3	2
History of HPV vaccination	No	No	No
History of smoking	No	No	No

^a^Q denotes patients identified from the questionnaire cohort; O denotes patients identified from outpatient clinical records; QO denotes patients identified in both sources

^b^HSIL: high-grade squamous intraepithelial lesion

^c^LSIL: low-grade squamous intraepithelial lesion

#### Clinical characteristics of All HPV-positive patients

Across the questionnaire and outpatient datasets, three patients tested positive for high-risk HPV. The first, aged 34, had undergone maternal vaginal mucosal transplantation 12 years earlier and was positive for non-16/18 h-HPV (type unknown), with HSIL cytology. She had two sexual partners, inconsistent condom use and intercourse three to five times per week. The second, aged 33, had undergone dilation and was positive for HPV 68 and 81, with LSIL cytology. She consistently used condoms, had three partners and had intercourse less than once per month. The third, aged 32, had a biological graft one year earlier and was positive for non-16/18 h-HPV (type unknown), with normal cytology. She had been sexually active for five years, had two partners, had intercourse one to three times per month and did not use condoms. None of the three had been vaccinated or smoked (Table 3).

## Discussion

Human papillomavirus may also affect individuals without a cervix, as HPV-related conditions—including genital warts, vaginal intraepithelial neoplasia, vulvar intraepithelial neoplasia, and vaginal cancer—have been reported among individuals with a neovagina [9]. Nevertheless, our study revealed a striking paradox: although 75.8% of MRKH patients recognised the importance of HPV vaccination, actual screening and vaccination rates were very low (11.5% and 18.5%). The vaccination rate was even lower than the 35.3% reported by Jessica Y. Shim et al. [10] This gap likely reflects barriers such as cost, misinformation, limited access, and the sensitivity of HPV-related topics. China has now launched a free HPV vaccination programme for girls aged ≥ 13 born after November 20, 2011, started in November 2025, and some regions already offer additional subsidies [11]. A lower-cost domestic 9-valent vaccine has recently become available, but financial and regional disparities continue to affect access. As vaccination programmes expand, prioritising vulnerable groups such as MRKH patients—especially for the 9-valent vaccine—may help improve coverage and reduce the risk of HPV-related neoplastic disease.

This study is also the first in China to investigate HPV infection among women with MRKH. HPV positivity was 11.1% in the questionnaire cohort and 7.4% in outpatient records. For comparison, a 2025 screening study in Xinjiang reported HPV positivity of 9.1% and 11.2% among women aged ≥ 25 [12], suggesting that HPV infection can occur in women with MRKH syndrome after neovagina reconstruction and should not be overlooked. Moreover, given the low awareness and uptake of HPV screening, the true rate in the MRKH population may be substantially underestimated. International data are limited to two German studies reporting HPV positivity rates of 2.9% and 22.0%, showing wide variability likely due to small sample sizes and differing sexual behaviours, as Chinese women generally report more conservative sexual activity (Table 4). Additional case reports described HPV-positive MRKH patients [6, 13], and HPV-related lesions in transgender individuals with neovagina [14]. Overall, current evidence indicates that HPV infection in the MRKH population has been largely overlooked and warrants greater clinical and research attention.

**Table 4:** Review of reported HPV infection rates in patients with MRKH syndrome

Author and year	Country	Sample size	Number of HPV-positive samples	HPV positive rate
Rall K et al., 2014[18]	Germany	240	7	2.9%
Rall KK et al., 2019[5]	Germany	186	41	22.0%
Questionnaire survey in this study^a^	China	18	2	11.1%
Outpatient investigation in this study^a^	China	27	2	7.4%

^a^HPV-positive rates are calculated based on the tested sub-populations in each study, rather than the overall MRKH population

The study tried to examine the potential influence of surgical technique on HPV outcomes, but no significant correlation was found, possibly due to the small sample size of the tested patients. Previous research has demonstrated alterations in the vaginal microbiota following MRKH surgery [15]. In addition to factors such as smoking and sexual activity levels, methods of neovaginal reconstruction, the composition of the vaginal microbiome, and the presence of vaginitis may also be associated with susceptibility to HPV infection. This observation warrants further investigation in larger cohorts to elucidate the relationship between surgical reconstruction methods and HPV-related morbidity more clearly.

In our outpatient cohort of 1,756 MRKH patients, only 30 received HPV and TCT testing, indicating a clear lack of clinical attention to HPV screening in this population. This is concerning given the oncogenic risk of persistent high-risk HPV infection [16]. A Finnish case further underscores this issue: a patient who underwent Davydov vaginoplasty in 2020 was later found to have HPV 16, VAIN 2 and a benign pelvic leiomyoma [13]. The rarity of MRKH syndrome may lead gynecologists to overlook the possibility of HPV infection in sexually active women after neovagina reconstruction [17]. Our findings highlight the need to increase gynecologists' awareness of HPV infection in this population and to strengthen HPV-related health education for patients. Particular emphasis should be placed on promoting HPV vaccination and condom use.

During outpatient treatment, it was observed that some patients primarily associated condoms with contraception, as indicated by the Chinese term for condom, which literally means "contraceptive sheath". Consequently, these patients mistakenly believed that condoms were only designed to prevent pregnancy and frequently overlooked their critical role in preventing sexually transmitted diseases. This led to the misconception that condoms were unnecessary. Furthermore, high-risk sexual behaviours, such as having multiple sexual partners and infrequent condom use, were prevalent among the study participants, mirroring the findings of Frega et al. [6]. Notably, 34% of sexually active patients reported having two or more partners, which significantly elevates the risk of persistent HPV infection and subsequent neoplastic transformation.

In China, HPV is closely associated with cervical cancer. Given that patients with MRKH syndrome lack a cervix, some patients mistakenly believe that they are immune to HPV infection and do not require vaccination against it. In our outpatient study, three patients did not undergo HPV and TCT testing despite being prescribed it, reflecting a lack of awareness of disease prevention among MRKH patients. Based on the findings of our questionnaire survey, the lack of awareness of HPV infection and vaccination among MRKH patients highlights the need for health education in this group. It is particularly important to emphasise the need for consistent condom use during sexual activity. Such counselling could play a pivotal role in mitigating persistent HPV infection and its potential progression.

So far, Germany is the only country that has reported HPV infection rates in the MRKH population twice. This study is the third report in the world and the first one in China, filling a research gap. We also conducted an investigation from the perspective of patients' awareness of HPV protection, highlighting the significant gap in health education for this population. This is currently the largest survey on HPV prevention measures among patients with MRKH. These constitutes strengths of our study.

While several limitations should also be acknowledged. First, the small size of the HPV-tested cohort (*n* = 18, *n* = 27) restricted the statistical power of the prevalence findings. Second, in the questionnaire survey, reliance on self-reported data may introduce recall bias. Third, the questionnaire cohort was recruited from a managed WeChat support group established and overseen by senior clinicians at a tertiary referral centre. Although this approach facilitated recruitment of a relatively large number of patients with this rare condition, it may have introduced selection bias. Patients who actively participate in a specialised online support group may be more clinically engaged, more health-conscious and more familiar with MRKH-related health issues than patients without access to specialist care or online patient networks. Therefore, the relatively high level of self-reported HPV-related awareness observed in this study may not be generalisable to the broader MRKH population in China, particularly patients from rural areas or non-specialised clinical settings. Finally, due to the limited number of HPV-positive patients (*n* = 3), it is not possible to discuss the relationship between infection and surgical methods, vaginal microecological disorders, and other factors. Further studies with larger sample sizes, objective outcome measures, and correlation analyses are required to confirm the HPV infection rate and susceptible factors in MRKH patients.

## Conclusions

Low HPV vaccination coverage among patients with MRKH represents a critical gap in primary prevention and may substantially increase their risk of HPV-related morbidity. This vulnerability is further compounded by insufficient preventive measures, including unprotected sexual behaviour and low screening uptake. To address these shortcomings, there is an urgent need for multidisciplinary care models that integrate subsidised HPV vaccination, education on HPV risk in the neovagina, and comprehensive sexual health education. Such an approach could decrease high-risk HPV infections and ultimately reduce long-term oncogenic risks in this vulnerable population.

## Supplementary Information

Supplementary Material 1.

## Data Availability

Researchers who want access to datasets for replication should apply to azhai0101@163.com

## Abbreviations

MRKH: Mayer-Rokitansky-Küster-Hauser
HPV: Human papillomavirus
LSIL: Low-grade squamous intraepithelial lesion
HSIL: High-grade squamous intraepithelial lesion
VAIN: Vaginal intraepithelial neoplasia
PUMCH: Peking Union Medical College Hospital
CAIS: Complete androgen insensitivity syndrome
VAIN 2: High-grade intraepithelial lesion
