# Supplementary material for: Human papillomavirus awareness and prevention practices among patients with Mayer–Rokitansky–Küster–Hauser syndrome after neovagina reconstruction: a cross-sectional study
Source: BMC Womens Health. 2026 Jul 25;26:538. doi: 10.1186/s12905-026-04704-8 (PMC13685275; doi:10.1186/s12905-026-04704-8)
Supplement: Supplementary file 1 [file 12905_2026_4704_MOESM1_ESM.docx]

**Questionnaire on HPV Testing**

This questionnaire aims to investigate the level of understanding among MRKH girls regarding HPV virus prevention and screening. The personal information of yours will not be disclosed. Based on the summary of the questionnaire information, the HPV infection status of the MRKH group will be reported, and a plan for HPV screening and prevention within the MRKH group will be proposed.

1. Your Age (in years)

_________________________________

2. Do you smoke?

○Yes

○No

3. Have you undergone cervical cancer screening? (i.e., HPV, TCT tests)

○Yes

○No (Please skip to Question 8)

4. When was your most recent HPV or TCT test?

○Within the last year

○More than 1 year but less than 3 years ago

○More than 3 years but less than 5 years ago

○More than 5 years ago

5. Was your HPV result positive?

○Positive (infected)

○Negative (not infected)  (Please skip to Question 7)

○Can't remember/can't find the result (Please skip to Question 7)

6. Which HPV type was positive? (e.g., HPV 16, HPV 18, or other positive types listed on the report)

_________________________________

7. What was your TCT result?

□NILM (No intraepithelial lesion or malignancy)

□ASCUS (Atypical squamous cells of undetermined significance)

□ASC-H (Atypical squamous cells, cannot exclude high-grade squamous intraepithelial lesion)

□LSIL/ CIN I/ VaIN I (Low-grade squamous intraepithelial lesion)

□HSIL/ CIN II-III/ VaIN II-III (High-grade squamous intraepithelial lesion)

□AGC (Atypical glandular cells)

□Squamous cell carcinoma or adenocarcinoma

□Other, please specify _________________

□Can't remember/can't find the result

8. Have you received the HPV vaccine injection?

○Yes

○No (Please skip to Question 10)

9. Which vaccine did you receive?

○Imported 9-valent vaccine (Merck's Gardasil 9)

○Imported 4-valent vaccine (Merck's Gardasil)

○Imported 2-valent vaccine (GSK's Cervarix)

○Domestic 2-valent vaccine (Wantai's Cecolin, Walvax)

○Other, please specify _________________

*After completing this question, please skip to Question **11**.

10. Are you willing to receive the HPV vaccine in the future?

○Yes, I want the 9-valent vaccine

○Yes, I want the 4-valent vaccine

○Yes, I want the 2-valent vaccine

○No

11. Do you think it is necessary for MRKH girls to receive the HPV vaccine?

○Yes

○No

12. Do you think MRKH sisters can be infected with HPV?

○Yes

○No

13. Have you undergone vaginal reconstruction surgery?

○Yes

○No (Please skip to Question 15)

14. What type of surgery was performed?

○Biological patch vaginoplasty

○Peritoneal vaginoplasty

○Intestinal vaginoplasty

○Oral mucosa vaginoplasty

○Other, please specify_________________

*After completing this question, please skip to Question **16**.

15. Have you undergone or are you currently undergoing dilation therapy?

○Yes

○No

16. Have you ever had sexual intercourse?

○Yes

○No (Please skip to the end of the questionnaire and submit)

17. Do you always use condoms during the whole sexual intercourse?

○Yes

○No

18. How long have you been sexually active?

○Started within the last year

○More than 1 year but less than 3 years

○More than 3 years but less than 5 years

○More than 5 years

19. How often do you have sexual intercourse?

○More than 5 times per week

○3-5 times per week

○1-2 times per week

○1-3 times per month

○Less than once per month

20. How many sexual partners have you had to date?

_________________________________
